# Expression and reactivation of HIV in a chemokine induced model of HIV latency in primary resting CD4+ T cells

**DOI:** 10.1186/1742-4690-8-80

**Published:** 2011-10-12

**Authors:** Suha Saleh, Fiona Wightman, Saumya Ramanayake, Marina Alexander, Nitasha Kumar, Gabriela Khoury, Cândida Pereira, Damian Purcell, Paul U Cameron, Sharon R Lewin

**Affiliations:** 1 Department of Medicine, Monash University, Melbourne, VIC, Australia; 2 Centre for Virology, Burnet Institute, Melbourne, VIC, Australia; 3 Department of Microbiology, University of Melbourne, Melbourne, Australia; 4 Infectious Diseases Unit, Alfred Hospital, Melbourne, Australia

**Keywords:** Chemokines, HIV latency, resting CD4+ T-cells, viral RNA, HDACi

## Abstract

**Background:**

We recently described that HIV latent infection can be established *in vitro *following incubation of resting CD4+ T-cells with chemokines that bind to CCR7. The main aim of this study was to fully define the post-integration blocks to virus replication in this model of CCL19-induced HIV latency.

**Results:**

High levels of integrated HIV DNA but low production of reverse transcriptase (RT) was found in CCL19-treated CD4+ T-cells infected with either wild type (WT) NL4.3 or single round envelope deleted NL4.3 pseudotyped virus (NL4.3- Δenv). Supernatants from CCL19-treated cells infected with either WT NL4.3 or NL4.3- Δenv did not induce luciferase expression in TZM-bl cells, and there was no expression of intracellular p24. Following infection of CCL19-treated CD4+ T-cells with NL4.3 with enhanced green fluorescent protein (EGFP) inserted into the *nef *open reading frame (NL4.3- Δnef-EGFP), there was no EGFP expression detected. These data are consistent with non-productive latent infection of CCL19-treated infected CD4+ T-cells. Treatment of cells with phytohemagluttinin (PHA)/IL-2 or CCL19, prior to infection with WT NL4.3, resulted in a mean fold change in unspliced (US) RNA at day 4 compared to day 0 of 21.2 and 1.1 respectively (p = 0.01; n = 5), and the mean expression of multiply spliced (MS) RNA was 56,000, and 5,000 copies/million cells respectively (p = 0.01; n = 5). In CCL19-treated infected CD4+ T-cells, MS-RNA was detected in the nucleus and not in the cytoplasm; in contrast to PHA/IL-2 activated infected cells where MS RNA was detected in both. Virus could be recovered from CCL19-treated infected CD4+ T-cells following mitogen stimulation (with PHA and phorbyl myristate acetate (PMA)) as well as TNFα, IL-7, prostratin and vorinostat.

**Conclusions:**

In this model of CCL19-induced HIV latency, we demonstrate HIV integration without spontaneous production of infectious virus, detection of MS RNA in the nucleus only, and the induction of virus production with multiple activating stimuli. These data are consistent with *ex vivo *findings from latently infected CD4+ T-cells from patients on combination antiretroviral therapy, and therefore provide further support of this model as an excellent *in vitro *model of HIV latency.

## Background

Long-lived latently infected resting memory CD4+ T-cells persist in patients on suppressive combination antiretroviral therapy (cART) and are thought to be the major barrier to curing HIV infection [1-5]. Given the low frequency of latently infected memory CD4+ T-cells in vivo [5-9], robust *in vitro *models of HIV latency in primary CD4+ T-cells are urgently needed to better understand the establishment and maintenance of latency as well as identify novel strategies to reverse latent infection (reviewed in [10]).

We have previously demonstrated that latent infection can be established in resting memory CD4+ T-cells *in vitro *following incubation with the chemokines CCL19 and CCL21 (ligands for CCR7), CXCL9 and CXCL10 (ligands for CXCR3) and CCL20 (ligand for CCR6) [11,12]. These chemokines are important for T-cell migration and recirculation between blood and tissue [13-15], and we have proposed that the addition of chemokines *in vitro *to resting CD4+ T-cells may model chemokine rich micro-environments such as lymphoid tissue [11,16]. This model of chemokine-induced HIV latency is highly reproducible, leading to consistent high rates of HIV integration, limited viral production and no T-cell activation [11,12]; and it therefore provides a tractable model to dissect the pathways of how latency is established and maintained in resting CD4+ T-cells.

Latently infected resting CD4+ T-cells are significantly enriched in tissues such as the gastrointestinal (GI) tract [17,18] and lymphoid tissue [19]. *Ex vivo *analysis of these cells has demonstrated that despite detection of integrated HIV, spontaneous virus production does not occur [20]. There are multiple blocks to productive infection in infected resting CD4+ T-cells from patients on cART, including a block in initiation and completion of HIV transcription as well as a block in translation of viral proteins by the expression of microRNAs (reviewed in [21]]. In addition, a clear block in export of multiply spliced (MS) RNA from the nucleus to the cytoplasm has been demonstrated [22]. Infectious virus can be induced from resting CD4+ T-cells from patients on cART following stimulation *ex vivo *with mitogens such as phytohemaglutinnin (PHA) or phorbol myristate acetate (PMA); T-cell receptor activation using anti-CD3 and anti-CD28 [1,2]; or other stimuli such as IL-7 [23], IL-2 [23], the protein kinase C (PKC) activator prostratin [24,25], histone deacetylase inhibitors (HDACi) such as vorinostat [26,27], methylation inhibitors [28,29] or a combination of these approaches [25]. Ideally, reactivation of virus from *in vitro *models of HIV latency should also closely mimic *ex vivo *findings from patient derived CD4+ T-cells.

The main aim of this study was to examine whether there was any spontaneous viral production in our chemokine-derived model of latency, to identify the point in the virus life cycle where virus expression was restricted, and to identify activation strategies that induce virus production from these latently infected CD4+ T-cells. Our results demonstrated that there was no production of infectious virus in this *in vitro *model of HIV latency, and that the block to productive infection and response to activating stimuli closely mimic findings from latently infected CD4+ T-cells from patients on cART.

## Results

### Latency is established in CCL19-treated CD4+ T-cells following single round infection, and there is no evidence of spontaneous productive infection

**Figure 1:**
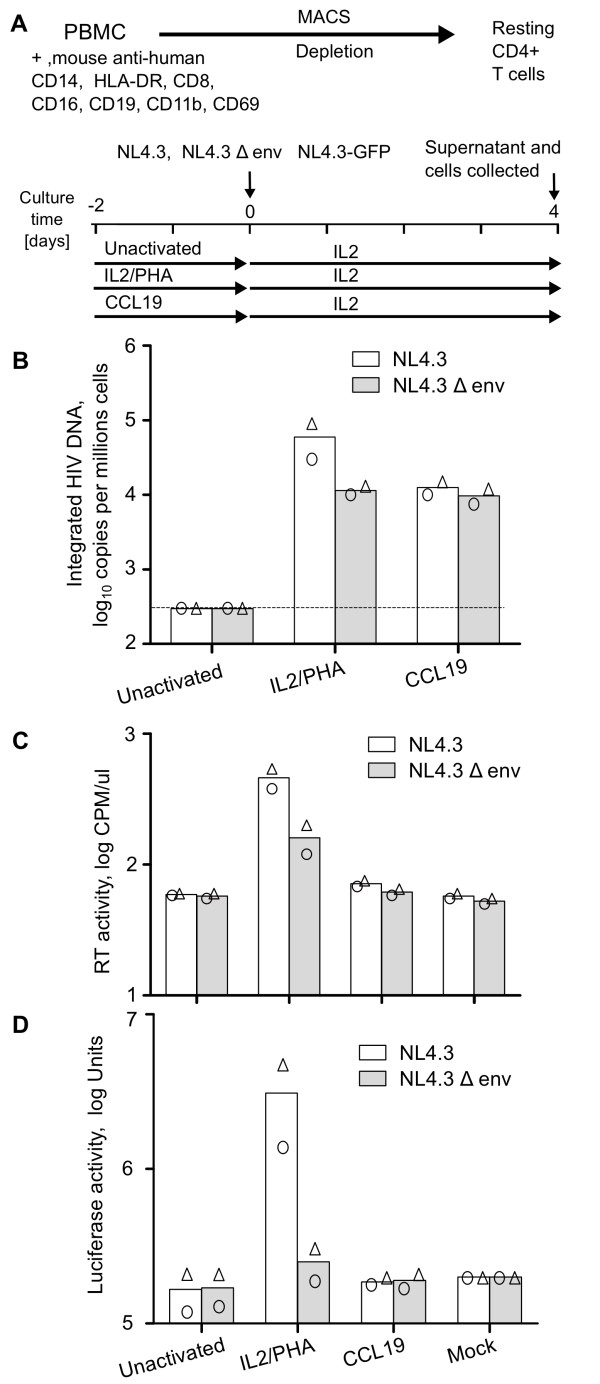
**High levels of HIV integration with minimal virus production in CCL19-treated infected CD4+ T-cells consistent with latent infection**. (A). Schematic diagram of the experimental protocol used for isolation of CD4+ T-cells and HIV infection. Resting CD4+ T-cells were cultured for 2 days with PHA/IL-2, CCL19 or without activation (unactivated). Cells were then infected with WT NL4.3, NL4.3Δenv or NL4.3-Δnef/EGFP or mock for 2 hrs and virus was washed off. The infected cells were cultured with media containing IL-2 (10 IU/mL) for 4 days. Infection of cells with WT NL4.3 (open bars) or NL4.3Δenv (grey bars) was quantified by detection of (B) integrated HIV DNA (C) RT activity (CPM/μl) in culture supernatant or (D) luciferase activity of supernatants using the TZM-bl indicator cell line. In all graphs, the mean (column) and individual data (open symbols) from two donors are shown.

We infected CCL19-treated CD4+ T-cells with WT NL4.3 and NL4.3Δenv to determine if spreading infection contributed to the high levels of integrated HIV observed following infection of CCL19-treated CD4+ T-cells. Consistent with our previous work [11,12], incubation of resting CD4+ T-cells with CCL19 followed by infection with WT NL4.3 resulted in high levels of viral integration and minimal production of RT in the supernatant, consistent with latent infection (Figure 1B and 1C). Infection with NL4.3Δenv also resulted in high levels of viral integration with levels similar to that observed following infection with WT NL4.3 (Figure 1B and 1C). As expected, infection of IL-2/PHA activated cells with NL4.3Δenv led to reduced RT production and a 10 fold reduction in integrated HIV. Integration of HIV was not observed following infection of unactivated resting CD4+ T-cells with either NL4.3 or NL4.3Δenv (Figure 1B and 1C). These data demonstrate that multiple rounds of infection did not contribute to high levels of integration observed in CCL19-treated infected CD4+ T-cells.

To determine if there was production of any infectious virus in CCL19-treated infected CD4+ T-cells, we infected cells with either WT NL4.3 or NL4.3Δenv (as described in Figure 1A) and collected supernatants at day 4 following infection. We then cultured these supernatants with the indicator cell line TZM-bl and assessed luciferase activity. Only the supernatant derived from IL-2/PHA activated CD4+ T-cells infected with WT NL4.3 led to an increase in luciferase activity consistent with production of infectious virus in these fully activated CD4+ T-cells (Figure 1D). No infectious virus was detected in supernatants from CCL19-treated or unactivated CD4+ T-cells infected with either WT NL4.3 or NL4.3Δenv (Figure 1D).

**Figure 2:**
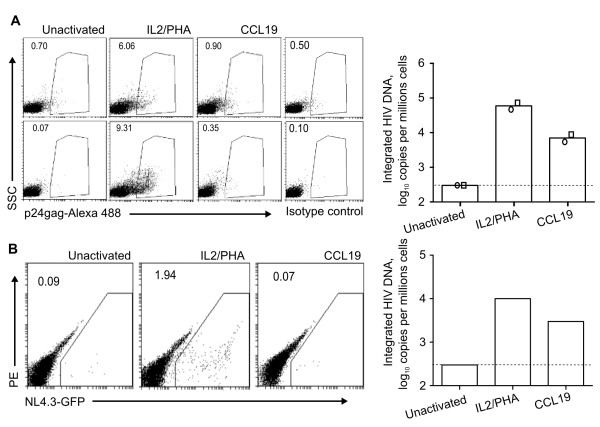
**No productive HIV infection in CCL19-treated infected cells**. Resting CD4+ T cells were cultured for 2 days with PHA/IL-2, CCL19 or without activation (unactivated). Cells were infected with NL4.3 or NL4.3-Δnef/EGFP for 2 hrs. The virus was washed off and the infected cells were cultured with media containing IL-2 (10 IU/mL) for up to 4 days. Productive infection was detected by flow cytometry of (A) intracellular HIV p24 (Alexa-488) following infection with NL4.3 (left panels; two representative donors). The right hand panels show the corresponding integrated copies of HIV per million cells for the same donors (as the mean of two donors (column) and as individual data (open symbols)). (B) Productive infection was detected by flow cytometry of EGFP expression following infection with NL4.3-Δnef/EGFP (one donor). The integrated copies of HIV per million cells are shown for this donor (right hand panel). SSC = side scatter.

The absence of productive infection was further confirmed by staining for intracellular p24 expression where we found that CCL19-treated infected CD4+ T-cells resulted in <1% p24-positive cells in contrast to IL-2/PHA activated infected CD4+ T-cells (mean p24 expression ~6-9%; n = 2; Figure 2A). Finally, following infection with NL4.3Δnef/EGFP of CCL19-treated and IL-2-PHA activated CD4+ T-cells, EGFP expression was 0% and 2% respectively (n = 1; Figure 2B).

Taken together, these experiments clearly demonstrated that in the presence of high levels of HIV integration in CCL19-treated infected CD4+ T-cells, there was no production of infectious virus as measured by infectivity of supernatants, p24 production or EGFP production consistent with latent infection.

### High level of MS RNA but low levels of US RNA in latently infected CCL19-treated CD4+ T-cells

**Figure 3:**
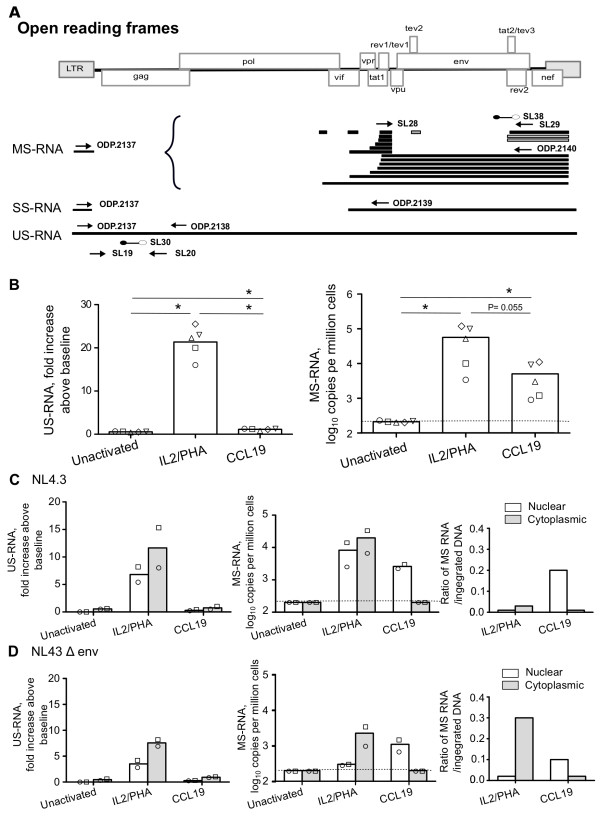
**High level of nuclear MS RNA in CCL19-treated latently infected CD4+ T-cells**. (A) Schematic diagram of the broad classes of HIV mRNA, including multiply spliced (MS) singly spliced (SS) and unspliced (US) HIV RNA (adapted from [56]). Location of primers and probes used for real-time PCR quantification of US, SS and MS RNA are shown. (B) Resting CD4+ T cells were activated with IL-2/PHA, CCL19 or unactivated and infected with NL4.3 or NL4.3Δenv. The fold increase in US RNA at day 4 post infection (relative to day 0; left panels) and the absolute copies per million cells of MS RNA (right panels) is shown for (B) total RNA following infection with WT NL4.3 (n = 5) and (C and D) nuclear (open) and cytoplasmic (grey) fractions following infection with (C) NL4.3 and (D) NL4.3Δenv. The mean (column) and individual data (open symbols) are shown. The ratio of MS RNA to integrated DNA for each donor is also shown (C and D, far right panel). The detection limit for US RNA and MS RNA was 200 copies/10^6 ^cells and is shown as dashed line for MS RNA. * = *P *< 0.05.

To identify the point in the virus life cycle following HIV integration where virus expression was restricted in this model of CCL19-induced HIV latency, we next examined expression of US and MS RNA (location of primers are summarised in Figure 3A). The mean fold increase of US RNA (expression at day 4 compared to day 0) following infection of PHA/IL-2 activated, CCL19-treated and unactivated CD4+ T-cells was 21.1, 1.1 and 0.5 fold respectively (n = 5; p < 0.05 for all comparisons; Figure 3B). We measured the fold change in US RNA in these experiments because US RNA was always detected at baseline i.e. immediately following virus removal by washing (average 3,700 copies/million cells in all conditions) which we assumed was US RNA in the viral inoculums that had adhered to the surface or was endocytosed in the CD4+ T-cells. When we adjusted for the amount of integrated HIV DNA in the same experiment for each condition, the mean US RNA: integrated DNA ratio was 0.25 and 0.08 for PHA/IL-2 activated and CCL19-treated infected CD4+ T-cells respectively (n = 5).

The mean copy number of MS RNA in IL-2/PHA activated, CCL19-treated and unactivated CD4+ T-cells infected with WT NL4.3 was 56,000, 5,000 and <200 copies/million cells respectively (n = 5; Figure 3B). The levels of MS RNA were not significantly different between the IL-2/PHA and CCL19 activated cells (*P = *0.055). However, MS RNA was significantly higher in both infected IL-2/PHA and CCL19 treated cells when compared to unactivated cells (*P *= 0.01). When we adjusted for the amount of integrated HIV DNA in the same experiment, the mean MS RNA: integrated DNA ratio was 0.1 and 0.6 for PHA/IL-2 activated and CCL19-treated infected CD4+ T-cells respectively (n = 5; Figure 3). We also examined production of 4 kb singly spliced (SS) RNA (primers 0dp 2137 Universal forward and 0dp 2139 reverse; Figure 3A) and found high level expression in IL-2/PHA activated infected CD4+ T-cells, and low levels in CCL19-treated infected CD4+ T-cells while SS RNA was not detected in unactivated CD4+ T-cells (data not shown). Using a different set of primers to measure US and MS RNA (0dp 2137, 0dp 2138, 0dp 2139, and 0dp2140, Table 1) with two different donors, we further confirmed our findings of no production of US RNA but high level production of MS RNA in CCL19-treated infected CD4+ T-cells (data not shown).

**Table 1:** Names, description and sequences of the primers and probes used for the real-time qPCR for HIV integrated DNA, US RNA, MS RNA, and SS RNA

Name	Description	Sequence
MH535	1^st ^rd forward 3'LTR	5' -AACTAGGGAACCCACTGCTTAAG-3'

SB407	1^st ^rd reverse Alu	5' -TGCTGGGATTACAGGCGTGAG-3'

SL75	2^nd ^rd forward	5' -GGAACCCACTGCTTAAGCCTC-3'

SL76	2^nd ^rd reverse	5' -GTCTGAGGGATCTCTAGTTACC-3'

SL72	beacon	FAM-CGGTCGAGTGCTTCAAGTAGTGTGTGCCCGTC CGACCG-TAMRA-3'

SL19	US forward	5' - TCTCTAGCAGTGGCGCCCGAACA-3'

SL20	US reverse	5' - TCTCCTTCTAGCCTCCGCTAGTC-3'

SL30	US beacon	5' FAM-CGGGAG TACTCACCAGTCGCCGCCCCTCGCC CTCCCG (Dabcyl) 3'

SL28	MS Forward	5' - CTTAGGCATCTCCTATGGCAGGAA - 3'

SL29	MS reverse	5' - TTCCTTCGGGCCTGTCGGGTCCC - 3'

SL38	MS RNA beacon	5' GGGCCT TCTCTATCAAAGCAACCCACCTCC AGGCCC -3'

0dp 2137	Universal forward	5' CGCACGGCAAGAGGCAGG-3'

0dp 2138	US reverse	5' CCCGCTTAATACCGACGCTCTCG-3'

0dp 2140	MS reverse	5' GTCGGGTCCCCTCGGGATTGG-3'

0dp 2139	SS reverse	5' AGGTTGCATTACATGTACTACTTACTGCTT-3'

LTR = long terminal repeat; US = unspliced HIV RNA; MS = multiply spliced HIV RNA; SS = singly spliced HIV RNA

To further determine why MS RNA production in CCL19-treated infected CD4+ T-cells did not lead to efficient expression of US RNA, we examined both US and MS RNA in cytoplasmic and nuclear fractions from infected IL-2/PHA activated, CCL19-treated, and unactivated CD4+ T-cells. Both US and MS RNAs were detected in the cytoplasmic and nuclear fractions in IL-2/PHA activated infected CD4+ T-cells (Figure 3C). As expected, US RNA was low in both cytoplasmic and nuclear fractions in CCL19-treated and unactivated infected CD4+ T-cells. MS RNA was almost entirely localized to the nucleus in CCL19-treated infected CD4+ T-cells, and was not detected in either fraction in unactivated CD4+ T-cells (Figure 3C and 3D). The ratio of nuclear MS RNA to integrated DNA in IL-2/PHA-activated and CCL19-treated infected cells was 0.02 and 0.15 respectively (n = 2; Figure 3C and 3D).

Taken together, these data demonstrate that in CCL19-treated infected CD4+ T-cells, production of MS RNA occurs, but there is no MS RNA detected in the cytoplasm, similar to descriptions of resting CD4+ T-cells from HIV-infected patients on cART [22].

### Virus production from latently infected CCL19 stimulated cells

**Figure 4:**
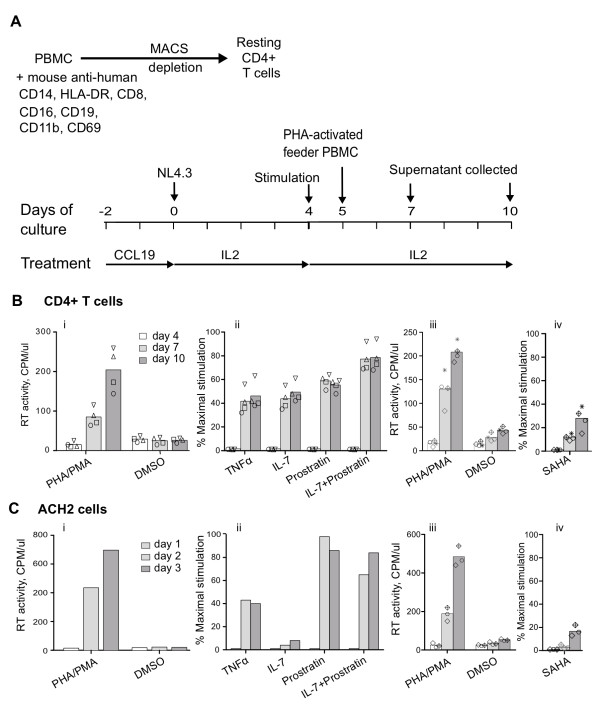
**Virus production from latently infected CCL19 stimulated cells**. (A) Schematic diagram of the experimental protocol used for infection and restimulation of CCL19-treated resting CD4+ T-cells. Resting CD4+ T-cells were cultured for 2 days with CCL19 and infected with NL4.8 and then restimulated with different activation agents at day 4 post- infection (PI). PHA-stimulated activated PBMCs were added at a ratio of 2:1. The cultures were maintained in IL-2 alone. Supernatant was harvested at day 7 and 10 PI to detect RT activity. (B) RT activity (CPM/μl) was measured following incubation of CCL19-treated infected CD4+ T-cells with (i) PHA/PMA (maximal stimulation) or with DMSO, (ii) TNFα, IL-7, prostratin and the combination of IL-7 with prostratin (expressed as percentage of maximal stimulation), (iii) PHA/PMA or DMSO (from different donors for vorinostat (SAHA) experiments) or (iv) vorionostat (SAHA) expressed as percentage of maximal stimulation (from (iii)). The mean (column) and individual data (open symbols) from four donors are shown. (C) The latently infected cell line ACH2 was also incubated with the same stimuli as in (B) and supernatant collected at day 1, 2 and 3 post stimulation. The mean (column) and individual data (open symbols) from three separate experiments, following activation with vorinostat (SAHA) are shown.

Finally, we used our model of CCL19-treated latently infected CD4+ T-cells to determine if cellular activators and the HDACi vorinostat could induce viral expression and compared the response to the latently infected T-cell line ACH2 (Figure 4B). The mean (range) production of RT (expressed as a percentage of maximal stimulation with PHA/PMA) following stimulation with TNFα was 38% (32-56%); IL-7 was 43% (35-55%); prostratin was 57% (51-64%); vorinostat was 12% (9-15%) day 7 post infection (day 3 post stimulation) with higher levels of RT production by day 10 post-infection following TNFα, IL-7 and vorinostat, but not following prostratin (n = 4; Figure 4B). The combination of IL-7 and prostratin, resulted in the highest levels of RT production (76% (68-92%)) which in one donor approached that of the maximal stimulation with PHA/PMA (Figure 4B, inverted triangles). In the ACH2 cell line, all stimuli led to induction of virus expression except that there was no response to IL-7 (Figure 4B).

## Discussion

We have previously established an *in vitro *model of HIV latency following incubation of resting CD4+ T-cells with the CCR7 ligands, CCL19 [11,12]. We have shown here that, in this *in vitro *model of HIV latency, there was no spontaneous production of infectious virus and that the block in the virus life cycle and the response to activating stimuli closely mirrors findings by other groups in *ex vivo *resting CD4+ T-cells from HIV-infected patients on cART [20,22,23,25].

We found that in CCL19-treated latently infected cells MS RNA was detected in the nucleus, but not in the cytoplasm, in contrast to PHA/IL-2 activated infected cells where MS RNA was detected in both nucleus and cytoplasm. MS RNA encodes the positive regulators Rev and Tat that are crucial for the efficient expression of US RNA in the cytoplasm [30,31]. Therefore, the lack of US RNA expression and viral production in CCL19-treated infected CD4+ T-cells may be explained by the absence of MS RNA in the cytoplasm. The absence of MS RNA in the cytoplasm could potentially be secondary to a block in nuclear export of viral mRNA or destruction of MS RNA in the cytoplasm. We were unable to distinguish between these two possibilities; however, others have previously described that in CD4+ T cells from patients on cART, there is a block in export of MS RNA to the cytoplasm secondary to low levels of polypyrimidine tract binding protein in resting CD4+ T-cells [22]. We have recently compared gene expression using Illumina microarrays in resting CD4+ T-cells with and without CCL19 [11], and found no difference in the expression of PTB in the presence or absence of CCL19 (data not shown). These data suggest that PTB may also be functional in this chemokine model of HIV latency, but further experiments will be required to demonstrate this directly.

Production of virus from CCL19-treated infected CD4+ T-cells was clearly demonstrated following activation with multiple different stimuli. The combination of IL-7 and prostratin resulted in the highest levels of RT production (Figure 4B). Prostratin stimulates HIV through PKC -mediated release of active nuclear factor κB (NF-κB) [24]. Previous studies have shown that inadequate or low nuclear levels NF-κB and nuclear factor of activated T cells (NFAT) may contribute to the maintenance of latency in resting CD4+ T-cells (reviewed in [32-34]). IL-7 has been shown to effectively induce HIV replication *ex vivo *in both CD8 depleted PBMCs and resting CD4+ T-cells from patients on cART [23]. IL-7 can activate both the PI3K and the STAT 5 pathways which could both potentially enhance virus transcription [35,36]. Activation of PI3K could increase virus transcription via enhanced production of NF-kB [37-39] while phosphorylated STAT5 has been shown to bind and transactivate viral transcription in *ex vivo *primary CD4+ T-cells; in the HeLa cell line co-transfected with STAT5 expression vectors and an HIV LTR construct that expresses firefly luciferase construct; and in the latently infected cell line (U1) [34,40,41]

IL-7 may also potentially contribute to the maintenance of HIV latency via homeostatic proliferation of resting CD4+ T-cells [5], but proliferation alone would not explain our findings that IL-7 can induce virus production from latently infected cells [42]. Furthermore, we found that IL-7 alone had no effect on T-cell proliferation of purified resting CD4+ memory T-cells which were used in this model, as measured by Ki67 staining and dilution of carboxyfluorosceinsuccinate (CFSE) (data not shown). The exact mechanism of action of IL-7 in our CCL19-induced model of latency remains unclear.

TNFα resulted in quite potent virus reactivation in our model which is consistent with findings in latently infected primary CD4+T cells that were transduced with the prosurvival molecule Bcl-2 [43] and in multiple latently infected cell lines [44,45]. In contrast, in another primary latency model using non-polarised cells that were activated, infected and allowed to rest, TNFα did not result in any virus reactivation [6]. In these two previous studies using primary T-cell models of latency, a similar concentration of TNFα, 10 ng/ml, was used as we have used in this study although the response rates were quite different with a percent maximal stimulation of 40%, 20% and 0% in our, the Yang [43] and Bosque [6] models respectively. The differences in detection of reactivation are unlikely to be explained by the frequency of latently infected cells as in our model of chemokine induced latency, on average 1% of cells contain integrated DNA, which is similar to the Yang model [43] but is far lower than the frequency of latently infected cells using the Bosque model, where the frequency of latently infected cells approached 30-50% [6]. To our knowledge, reactivation of latent infection with TNF-a has not been assessed in resting CD4+ T-cells from patients on suppressive cART and these experiments would add further insight to our understanding of the currently available different models of latency in primary T-cells.

Others have demonstrated the synergism obtained by treatment with a combination of prostratin and the HDACi vorinostat in both a cell line and primary cell model of latent HIV infection [46]. Herein we also demonstrated the additive effects in activation of HIV replication by combining the PKC activator prostratin with IL-7. We have not yet evaluated the effects of IL-7 with other HDACi in this model, but this will certainly be of interest given the well known safety profiles of drugs such as IL-7 and vorinostat. Strategies that activate latent HIV in infected individuals on cART are likely to include combinatorial approaches and our model provides a robust tool for screening such approaches.

## Conclusions

In this model of CCL19-induced HIV latency, we demonstrated highly efficient integration of HIV and no spontaneous production of infectious virus. MS RNA was produced, but was not detected in the cytoplasm consistent with findings from resting CD4+ T cells from patients on cART. Furthermore, virus could be activated using multiple different stimuli previously shown to activate virus production *ex vivo *from resting CD4+ T-cells from patients on cART. These data provide further support to this model as an excellent *in vitro *model to study HIV latency and a useful tool to screen for novel compounds to reverse latency.

## Methods

### Isolation of CD4+ T cells

Peripheral blood mononuclear cells (PBMC) were isolated from buffy coats obtained from the Australian Red Cross Blood Service (Southbank, Australia). Resting CD4+ T cells were isolated by magnetic bead depletion and cell sorting using a cocktail of antibodies to CD19, CD11b, CD14, HLA-DR, CD16 and CD69, as previously described [11,12]. The purity of resting CD4+ T cells was routinely >95% when assessed by flow cytometry.

### HIV Plasmids, transfection, and infection

HIV infection was performed with either the CXCR4-using wild type (WT) virus NL4.3 or NL4.3 with enhanced green fluorescent protein (EGFP), inserted into the *nef *open reading frame [NL4.3-Δnef/EGFP] at amino acid position 75 at the aKpnI (Acc651) site (kindly provided by Damian Purcell, University of Melbourne, Melbourne, Australia) or envelope deleted NL4.3 pseudotyped virus (NL4.3-Δenv). 293T cells were transfected with the plasmids for NL4.3 or NL4.3-Δnef/EGFP according to the manufacturer's instructions (FuGene; Roche Diagnostics, Indianapolis, IN). NL4.3-Δenv was generated by co-transfection of 293T cells with plasmid DNA encoding a deletion from bp 6343 to bp 7611 in *env *(kindly provided by Damian Purcell), and the SVIII plasmid containing the *env *of NL4.3 [47] (kindly supplied by M Churchill, Burnet Institute, Melbourne, Australia). Culture supernatants containing each of the above viruses were concentrated over 20% sucrose gradients and assessed for reverse transcriptase (RT) activity as previously described [48].

Purified resting CD4+ T-cells were incubated with the chemokine CCL19 at 29 nM (R&D Minneapolis, MN), PHA (10 μg/ml; Sigma, St Louis, MO) combined with IL-2 (10 IU/ml, Roche, Indianapolis, IN) or left unactivated for two days before HIV infection. Infection was performed with virus at a concentration of 1 count per minute (CPM) per cell for 2 hr at 37°C. The cells were then washed and cultured in the presence of IL-2 (10 IU/ml) as previously described [11,12]. The method used for infection of resting CD4+ T-cells is summarised in Figure 1A.

### RNA and DNA extraction

Whole cells were lysed in Trizol (Invitrogen) and total RNA extracted according to the manufacturer's instructions (RNeasy Mini Kit, Qiagen). Nuclear and cytoplasmic RNAs were obtained as previously described [22]. Genomic DNA was extracted from infected cells using the DNeasy DNA extraction kit (Qiagen) according to the manufacturer's instructions.

### Quantification of HIV infection

Production of HIV was quantified by measuring the Reverse Transcriptase activity in cell culture supernatant at days 4, 7, and 10 post-infection as previously described [48] as well as by measuring integrated HIV DNA and RNA at day 4 post-infection using real-time PCR (iCycler, Bio-Rad, Hercules, CA). Integrated HIV DNA was quantified using a nested Alu-long terminal repeat (LTR) PCR as previously described [49,50]. In brief, we used standards that contained random integration sites as previously described [51]; all samples were run in triplicate and we used an additional control reaction that included the LTR primer alone [49,50]. Input DNA was normalized by quantification of the CCR5 gene by real-time PCR [52]. Detection of unspliced (US) and multiple spliced (MS) HIV RNA was performed as previously described [49,53]. To adjust for the total cellular RNA in each sample, relative copy numbers were normalized to 18S rRNA [49]. The location of all primers and probes used are summarised in Figure 3A and Table 1.

### Flow cytometry analysis

Expression of EGFP was determined 4 days following infection using flow cytometry. For analysis of intracellular p24, cells were permeabilized with a mild detergent and stained with a monoclonal antibody against HIV p24 followed by detection with Alexa Fluor 488 goat anti-mouse IgG (H + L, Molecular probes, Eugene, OR) and samples were analyzed by FACSCalibur flow cytometer (BD Biosciences) as previously described [6]. Briefly, 1.5 to 2.5 × 10^5 ^cells were fixed and permeabilized with Cytofix/Cytoperm during 30 minutes at 4°C. Cells were washed with Perm/Wash Buffer and stained with a 1:50 dilution of mouse anti-p24 antibody (183) in 100 μL Perm/Wash Buffer during 30 minutes at 4°C. Cells were washed with Perm/Wash Buffer and incubated with 1:100 Alexa Fluor 488 goat antimouse IgG (H + L) in 100 μL Perm/Wash Buffer for 30 minutes at 4°C. Cells were washed with Perm/Wash Buffer and samples were analyzed by flow cytometry. Results were analyzed using the Weasel Flow cytometry analysis software (v2.7, Walter and Elisa Hall Institute, Melbourne, Australia).

### Detection of HIV infection in cell lines

The indicator TZM-bl cell line (obtained through the AIDS Research and Reference Reagent Program, Division of AIDS, NIAID, NIH from Dr. John C. Kappes, Dr. Xiaoyun Wu and Tranzyme Inc.) is derived from the HeLa cell line, expresses high levels of CD4, CXCR4 and CCR5 on the cell surface and is stably transfected with the luciferase and β-galactosidase genes under the control of the HIV LTR. TZM-bl cells were cultured in DMEM supplemented with 10% cosmic calf serum (CCS) and penicillin/streptomycin. Activation of the HIV LTR was detected using a luciferase assay as previously described [54]. Briefly, TZM-bl cells were placed at a concentration of 2 × 10^4 ^cells/well in 96-well flat-bottomed tissue culture plates and incubated for 24 h at 37°C, 5% CO_2_. The culture medium was replaced by culture medium containing 10μg/μl diethylaminoethyl-dextran (DEAE-dextran), and the cells were infected with 2-fold serial dilutions of culture supernatant from infected primary CD4+ T cells. After 48 h incubation at 37°C with 5% CO_2_, the medium was aspirated and 35 μl of cell culture lysis reagent (Promega, Wisconsin, USA) were added to each well. Five microliters of each sample were mixed with 25 μl of Luciferase Assay Substrate (Promega), and luminescence was measured using the FLUOstar OPTIMA multi-detector reader (BMG Labtech).

### Virus production from latently infected cells

To induce virus production, CCL19-treated latently infected CD4+ T-cells were incubated with PMA (10 nM, Sigma) and PHA (10 μg/mL, Sigma); TNFα (10 ng/mL, R&D); IL-7 (5 ng/mL, R&D); prostratin (5 μM, Sigma); a combination of IL-7 and prostratin; or the HDACi vorinostat (suberoylhydroxamic acid (SAHA); 0.5μM Selleck Chemicals, Houston, TX), on day 4 post-infection. PHA-activated feeder peripheral blood mononuclear cells (PBMCs) were added 24 h after the activating stimulus to amplify virus replication so as to enhance detection of infection. Virus production was measured in supernatant by quantification of RT production on days 7 and 10 post infection (day 3 and 6 following stimulation). The strategy used for activation is summarized in Figure 4A. The same stimuli were also used with the latently infected cell line ACH2 [55], (kind gift from the National Institutes of Health (NIH) Reagents Repository) and supernatants collected at day 1, 2 and 3 post-stimulation. In both latently infected CCL19-treated cells and in the ACH2 cell line, a combination of the mitogens PHA and PMA was used to achieve maximal viral production as measured by RT in supernatant. The viral production induced by TNFα, IL-7, prostratin, a combination of IL-7 and prostratin or vorinostat was then expressed as a percent of the maximal stimulation.

### Statistical analysis

Differences between experimental conditions were analysed using the Students t-Test or Mann Whitney non-parametric statistics (Graphpad prism version 3, Graphpad, Loa Jolla, CA). p < 0.05 was considered significant.

## Competing interests

The authors declare that they have no competing interests.

## Authors' contributions

SS and FW performed the majority of the experiments. SR, CFP, NK, GK and MA contributed to some specific experiments. DP provided reagents and contributed to the analysis of the data and helpful discussions. SS, FW, PUC and SRL designed the study. SRL supervised all aspects of the experimental work. All authors reviewed the final data and manuscript.
